# Observations on the Treatment of Bronchocele

**Published:** 1834-07-01

**Authors:** James Reid


### *Observations on the Treatment of Bronchocele.*

By James Reid, Esq., m.r.c.s.*

It has been the opinion of some authors that this disease is not of sufficient importance to require any particular method of treatment, as it produces deformity merely, unaccompanied by danger. Many of our own writers on this subject affirm, that they have met with no cases of goitre which terminated fatally. Gooch, for instance, who paid great attention to this disease, says he never knew life to be endangered by it, even when of large size. An American author, Dr. Gibson, affirms, too, that "few if any cases are on record of death from suffocation in this complaint." This is quite contrary to the facts which have fallen under my own observation; four cases of death from suffocation in this disease having come under my notice in the space of a year. Apoplexy occurs even more frequently, owing to the pressure of the tumor on the large veins of the neck. Numerous cases of fatal termination are detailed, too, in the continental journals; and, if we include those which, from their urgent and distressing symptoms, have (from being neglected at an early stage,) required a surgical operation (so often attended by fatal results,) we shall come to a far different conclusion. Sedillot, in his Journal de Medicine, relates the case of a boy, æt. fourteen, in whom the thyroid gland, without any assignable cause, enlarged most rapidly, and caused death by suffocation. Mr. Bromfield's death, I am informed, was owing to a similar cause, and another late member of the council of the College of Surgeons had a large bronchocele, chiefly affecting the right lobe, producing great fulness in the head and difficult respiration: in the latter case, however, at the age of sixty-five, part of the tumor put on a livid colour, and sloughed to about the size of an orange, which reduced him very much, but eventually was the cause of its entire disappearance.

The great danger to be apprehended from bronchocele arises, then, either from its large size and firm texture pressing on the neighbouring parts, (especially where, from the rapid

* Extracted, with some alterations, from an unpublished Essay which gained the Jacksonian prize in 1829.

increase of the tumor, the latter have not had time to accommodate themselves to the change,) or from the direction the tumor takes, encircling the trachea, even when not of large dimensions, as I have elsewhere remarked, in which cases the functions of respiration and deglutition are soon impeded:* should the disease partake of a scirrhous character, the danger is of course more imminent. Although Petit and others have related cases in which abscesses of a mild chronic nature had gradually formed in the enlarged gland, and dispersed it, still in some others this very circumstance has been attended by fatal results; an opening having been formed between its posterior portion and the trachea, and instant suffocation following.

The difficulty of effecting a cure in bronchocele was much felt by the ancients; at the present day, however, we possess remedies which will in a great majority of instances permit us to promise if not a total cure, still a great diminution of the tumor, and its accompanying distressing symptoms; in others they will fail in lessening the size of the gland, but yet prevent its further progress.

The two classes of bronchocele, accidental and endemic, require precisely the same mode of treatment, and, in speaking hereafter of any remedies, I shall consider them as applicable to either species. A very numerous list might be presented which have been held in repute at different periods, but I will mention among the principal ones only the internal use of soda, crabseyes, muriate of barytes, cicuta, belladonna, digitalis, and (the only one which has obtained any lasting credit) burnt sponge. Innumerable external remedies have been likewise recommended, such as friction with different stimulating oils, muriate of ammonia, mercurial ointment, compression, blisters, &c. Richter and Foderé found sulphuret of potass prove of service; while Prosser mentions a case in which chewing tobacco had removed the tumor, but at the same time he says he does not recommend the remedy to ladies, for fear of their gaining a habit of *quidding*, and not being able to leave it off. Diuretics have been praised by Gerard; whilst Dr. Gibson has found more benefit from the use of cicuta in bronchocele, than from any other remedy. The famous Coventry recipe, which formerly was held in such high estimation, consisted of ten grains of burnt sponge, with the same quantity of burnt cork and pumice-stone, made into a lozenge or bolus. The substances made use of by the ancients in the treatment of this complaint were various, and

* Vide Case.

of the most fanciful description; medicines composed of snakes, worms, &c. internally, and amulets, charms, and other external applications, are fully described by different Greek and Latin authors. "Bibe quotidie (says Johannus Agricola, for instance,) ex cranio hominis et inde struma evanescit;" but these remedies will be found amply and very uselessly discussed by several modern authors.

This tumor formerly came under that class of disorders which in our own country, as well as in France, were presented for the royal touch.

The treatment of bronchocele may be divided into three kinds, prophylactic, therapeutic, and chirurgical. The first will consist in avoiding, as much as possible, all the probable causes which influence its growth, especially in endemic goitre, such as removing from the district in which it was acquired; and this alone has been found sufficient to cure it in many instances: thus Foderé, who was himself affected by this disease till the age of fifteen, lost it by quitting Switzerland, but it returned when he afterwards went to reside at Strasbourg, although he was at that period advanced in years. The patient should be cautioned not to dwell in humid or ill-ventilated spots; the neck should be kept warm, and he should refrain from exercising the voice strongly, as in singing, reading aloud, or any other violent exertion. The state of general health should be particularly attended to, before commencing any specific mode of treatment.

The younger the patient, and the less firm and hard the tumor itself is, the greater likelihood is there of a complete cure; where it is of soft consistence, lobulated, and has not existed above three or four years, its removal is not generally difficult; but advanced age and long continuance of the growth are not so unfavorable as they were considered in the last century, when some writers narrated cases as singular in which the tumor had been dispersed after the age of twenty-five. Some are now on record in which those of twenty or thirty years' duration have been completely got rid of, under the employment of more modern remedies; amongst others one may be cited in which a large goitre was almost entirely removed in a female seventy years old.*

The therapeutic means to be employed in the cure of bronchocele consist, firstly, in the application of local remedies, either to detract blood from the vessels of the tumor, or to favour the increased action of its absorbents; and, secondly, in the exhibition of medicines supposed to act in a specific manner upon it.

* London Med. and Phys. Journal, August, 1823.

*Simple compression* seems sufficient in many recent cases to disperse the morbid swelling of the gland, and is a common procedure in the Alpine countries both of Europe and Asia, for this purpose. Flajanii, in his Collez. d'Osservaz. e Rifless. di Chirurg. vol. iii., relates several instances of successful treatment by compression combined with stimulating pledgets; whilst, in India and other countries, it has been found equally beneficial when applied at an early period of the complaint.

*Friction* alone has been thought by many modern authors to be still more efficacious, when regularly used; but stimulant oils or ointments will, I am persuaded, much increase its good effects. Electricity, blisters, and stimulating plasters, such as the Empl. Ammon. cum Hydrarg., have in some cases been attended with very good effect.

The topical abstraction of blood by the application of leeches to the tumor is a means which should always be used, before resorting to other more powerful remedies, as it will alone often produce a material diminution in its size. The difficult respiration, and uneasy sensations about the head and throat, are frequently lessened by this plan, and, combined with the employment of emollient cataplasms to the part, will much assist the effects of any specific remedies which we may afterwards employ internally, and predispose the cuticular pores to take up with greater rapidity those substances which we may judge it proper to apply locally. If the poultices are made slightly stimulant, by using equal parts of vinegar and water with linseed-meal, I have found their good effects increased. Blisters I have seldom found of much service, and for some time they prevent our using any other stimulating applications to the part.

Of all the specifics which have maintained a reputation at all, burnt sponge, as I have before remarked, is the principal, and the older medicinal remedies which acquired any fame are now proved to have contained it likewise. Dr. Henenschwand had recommended it unburnt, as equally efficacious, and not liable to the injurious effects which the former occasionally produced upon the stomach. It is a curious fact that Drs. Coindet, of Geneva, and Straub, of Berne, discovered about the same time, in 1820, unknown to each other, that its active principle resided in the iodine which it was found by them to contain, and accordingly, since that period, medical practitioners have used this latter medicine in various forms with remarkable effect not only in bronchocele, but in many other diseases. It is supplied abundantly by kelp, (the fucus saccharinus especially,) and was originally discovered in 1811 by M. Courtois, a soap-boiler, at Paris.

Iodine, from what experience I have had myself in its use, and from the mass of favourable evidence by native and foreign practitioners as to its powers, is, I am persuaded, a remedy of the highest efficacy, and that on which we must principally, if not altogether depend, in the treatment of this disease. It is most efficacious in recent cases, and in those where a soft elastic consistence is observable: in others, again, little or no effect can be produced by its use; but of course we are not to consider it an universal specific, but a remedy which will do much good in a great majority of cases. So marked is its power, that in some cases, where its use has been purposely intermitted, and another remedy substituted, the result has been, that no further diminution of the tumor took place till the iodine was resumed. In those cases, which are occasionally met with, where it is difficult to affect the individual with this medicine, I have found small bleedings from the arm hasten its effects in as marked a manner as those of mercurial preparations. Peculiar idiosyncrasies will, in some few instances, prevent its use altogether,—even the vapour of iodine producing very disagreeable effects in them: thus, M. Chevalier, having visited the manufactory of M. Curtois, on leaving it was seized with violent colic and heat of stomach, which were relieved by mucilage and laudanum. He was not aware of the cause which produced these symptoms till some time after, when, having broken a bottle of iodine, and been occupied in collecting it again, the same symptoms reappeared, and were relieved by a similar treatment. At all times, while the patient is gradually increasing the dose of this medicine, we should carefully watch its effects, and point out the periods when it may be prudent to intermit its use for a time. It occasionally produces very dangerous symptoms; and this was often the case on its first introduction among the inhabitants of the Alps; for some of those who were affected by goitre, hearing of the great efficacy of iodine, took it unadvisedly, until the frequent recurrence of serious consequences, resulting from its indiscriminate use, induced the Swiss government to issue an edict forbidding its sale, unless when prescribed by a physician.

These symptoms are more likely to follow its internal use than the local application of it to the tumor; and, on this account, I now prefer commencing with the latter.

In giving the tincture of iodine, or the solution of the hydriodate of potass, I have found more effect in commencing with very small doses, and slowly increasing them, than in administering it in sufficiently large quantities to affect the system very sensibly in a short time; and in this it bears some analogy to mercury.

I have rarely used the tincture; for I have found it more prone to produce alarming symptoms than the other preparations. Patients, too, often complained of its taste and heat, and in many instances it could not be at all tolerated by the stomach: on this account I for a long time was in the habit of using the hydriodate of potass dissolved in water, (forty grains to the ounce,) commencing with eight drops three times a day, and gradually increasing the dose to twenty. If good effects were to follow the employment of this remedy, I have generally found them to result from this quantity; and, if the dose was pushed further, the stomach and nervous system became deranged, without any corresponding diminution of the tumor. In conjunction with this medicine I almost invariably ordered the external application of an ointment, composed of ʒi. Hydr. Potass. to ℥i. of Axunge: a portion, the size of a nut, was well rubbed in the swelling, night and morning. I have never observed any disagreeable effects to arise from it; but, were they feared, they might be prevented by the combination of a little extract of opium, or acetate of morphia, with the ointment.

I have before mentioned the difficulty of affecting some individuals with iodine: thus Dr. Kennedy, in one case, beginning with two grains daily, increased the dose to eighteen grains, administering altogether 953 grains to the patient, without producing any sensible effect. Gendrin likewise relates the cases of two individuals of a family, whom he was unable to affect by iodine for the space of two months; but, by substituting one part of pure iodine with two of Phosph. Calcis, and rubbing in daily one grain of this on the gums and tongue, he at last succeeded.*

Of late I have found the form recommended by Lugol by far the best; viz. an aqueous solution of Iodine and Hydr. Potass.: the latter aiding greatly the solution of the former in water, and obviating, as Lugol says, the decomposition which the tincture of iodine undergoes when diluted, and the consequent deposition of the iodine upon the coats of the stomach. It resembles a mineral water when prepared in the following manner, and, although Lugol gives various formulæ of different strengths, in his work on Scrofula, this one will be found sufficient, I think, for practice; the medical attendant increasing the strength as he finds that it is necessary, or that the patient's stomach will bear it.

* Omodei Annali, 1829.

R. Iodinæ, gr. ⅛.:

Hydr. Potass. gr. iss.;

Aq. destill. ℥viij. M. to be taken in the course of the day.

The ointment which is applied to the tumor should consist of twelve grains of iodine, ℈iv. of Hydr. Pot., and ℥ij. of Axunge. Lugol has never seen any inconvenience follow the use of this form, and among one hundred females to whom he administered it in the Hôpital St. Louis, and whom he divided into three classes, the following was the result. The emaciated became stouter; the stout remained so; and the last class, who were of a medium size, rather gained flesh than lost it. Professor Brera, of Padua, in his "Saggio Clinico sull' Iodio," says that he has found iodine to emaciate the robust, but that it has the opposite effect upon spare habits. Dr. Gairdner's case has been the means of deterring many practitioners from using iodine to any extent; and this was the case with Dr. Fahnestock, who, by the frequent importunities of a patient affected by bronchocele, was at length induced to try it. The tumor in this case was large, pendulous, of a purple hue, and of eight years' standing, producing considerable pressure upon the trachea, and had resisted all other remedies. Under the iodine it began to decrease in fourteen days, and in the course of a month had entirely disappeared.* I have seldom found it to diminish the healthy glands; it appears to act almost solely on the morbid or adventitious structures, unless when injudiciously and rashly employed.

One remarkable proof of the efficacy of iodine in goitre is, that all the mineral springs in various parts of the world which have gained any great repute for the cure of the disease have been successively discovered to contain this substance. Thus, those of St. Genis, Aix in Savoy, and Castel Nuovo d'Asti, especially the last two, have been long famed for their power, and have, on careful analysis by Professor Cantu, of Turin, been proved to hold it in considerable quantity in solution under the form of an hydriodate; and he is of opinion that all sulphureous springs contain it. Angelini again has explained the long known efficacy of the waters of Sales, in Piedmont, by discovering iodine in them. We are informed by Georgi, that in the Cordilleras there is a spring, the use of which cures goitre in a very short time: "weim diese Leute *einige Wochen* das Wasser eines kleinen in die Anga bey Anginskoi fallenden Bächleins (Rutschai) trinken, so vergehen die Kröpfe, weinn sie nicht alt sind."† Iodine has been found

* American Journ. of Med. Sciences, 1829.

† Bemerkungen auf einer Reise im russisschen Reich, im Jahre 1772.

in the springs of Leamington, Cheltenham, and Bonnington, near Edinburgh; and, according to Balard and Pfaffin, exists in the waters of the Mediterranean and Baltic: it is not however unlikely, I think, that it may be found in all sea water.

Perhaps no specifics (if we except mercury in syphilis, and quinine in intermittents,) have met with stronger recommendations than iodine in the cure of bronchocele: both foreign and native practitioners concur in this opinion. Baup cured forty-five cases out of forty-six by its use; Irminger, fifty out of seventy; and Coindet, in twenty-two which he selected, cured all by iodine frictions. Bayle has found iodine a sovereign remedy in goitre; its efficacy, he says, surpassing that of any other. Mons. L. Janson, chirurgien en chef de Hôtel Dieu de Lyons, says, "nous avons retiré des effets avantageux et presque constans de la pommade de l'hydriodate de potasse;" he having, like many others, been obliged to desist from giving the tincture, owing to the irritation of the stomach which it produced. One case he relates of an individual, æt. twenty-three, who had a bronchocele consisting of three distinct lobes, each the size of an apple, where, after applying leeches and cataplasms, by the use of the ointment twice a day, it totally disappeared by the twenty-second day.* Dr. Coster, out of one hundred cases treated by iodine, says that two-thirds were cured; whilst Dr. Jahn considers it a most valuable medicine, and terms it "*un remede divin.*" Dr. Kolley, of Breslau, rid himself of a goitre by its use.

Many eminent practitioners of this country bear testimony likewise to the marked benefits derived in bronchocele from this medicine. Dr. Manson cured seventy-six out of 116, much relieved ten, and seventeen improved under its use. Dr. Forbes, of Chichester, and many others, concur in this favorable opinion; and Dr. Copland, in his valuable Dictionary, says, that of several cases of bronchocele which he has treated with iodine, there has not been one which was not cured, or remarkably improved by it. Mr. Bramley, who has had extensive opportunities of using it in India, out of 116 cases gives fifty-seven cured, fourteen nearly so, thirty-four much benefited, six partly relieved after two months' trial, and five altogether unsuccessful.

There is a fact connected with the administration of iodine which should be kept in mind, and which has, I believe, been observed by others as well as myself, viz. that it seems to be almost inert occasionally for a period, producing little or no effect upon the system; but, by intermitting its use for a short

* Observat. Cliniques.

time, on recommencing it the issue is most rapid and favorable. The good effects in some other cases seem to supervene a short time after discontinuing its employment, similarly to what we sometimes observe in the use of digitalis. In the foregoing remarks I do not allude to bronchocele only; but I have known the same occurrence to take place in ulcerated and scrofulous sores of the neck, which, though resisting the first, were healed as if by magic on the second trial of the iodine. Dr. Copland relates two cases strongly confirmatory of this fact.

Iodine I have found to possess great power over the uterine system; so much so, that I am now in the habit of employing it in some of the functional disorders of this organ, and it may on this account perhaps prove the more beneficial in many cases of bronchocele; the great sympathy between which and the uterus I have before made mention of. It is likewise a tonic of great power, and, when properly administered, invigorates the digestive organs.

When iodine has commenced its action upon this tumour, besides its gradual diminution, which may be most accurately remarked by regular measurements of the neck, the most pressing symptoms, such as the difficulty of breathing and swallowing, seem to subside first. Other symptoms, which might be easily mistaken at first as referable to some disease of the lungs, such as wheezing, hoarseness, palpitations, &c., now disappear gradually: as the improvement progresses, the voice becomes natural, the tumour more soft and elastic, and the patient gets rid of all the disagreeable feelings which had so long troubled him.

On the contrary, when, under its employment, the patient suffers from tremors, giddiness, debility, nausea, or vomiting of a glairy tenacious fluid, severe dragging pains of the stomach, attended by an indefinable sensation of hunger, which increases after meals, the tongue being furred and tremulous, and the pulse weak and rapid, we should immediately discontinue the medicine, and afterwards recommence with very minute doses. In fine, we should as carefully watch the effects of iodine on our patients, during the progress of the cure, as we are accustomed to do with digitalis or arsenic, and never allow them to continue it above four or five days in succession without seeing them, and thus judging whether it is prudent to continue its use.

There are some cases of bronchocele, which, either from long continuance, or from the peculiar structure of the tumour, withstand all medicinal applications, continue to increase in size, and at length are attended with most urgent symptoms, dangerous to the life of the patient: in these, and these only, recourse must be had to *surgical operations*. These are three in number, which, with their attendant good results and dangers, I will describe as briefly as possible.

*Seton.* The use of the seton, which was practised formerly, and afterwards recommended by Foderé, has been revived, within a few years past, by Dr. Quadri, of Naples, who relates several cases attended by successful results. He was led to adopt its use from the circumstance of a woman in Naples, afflicted with goitre, having, in a quarrel, received a wound in that part by a pair of scissors: it did not heal rapidly, and Dr. Q., who attended her at the hospital, observed that, owing to the discharge, a gradual diminution of the tumour took place, and it eventually disappeared. Simond, in his Tour through Italy, remarks, "I find that at length Dr. Q. found the practice dangerous, and had given it up." Many other surgeons, however, have performed the operation with happy results; among others, Dr. A. T. Thomson, Mr. Copland Hutchison, and Mr. James, of Exeter, though very severe symptoms followed in the last-mentioned case.

In the Edinburgh Medical and Surgical Journal for 1832, a case is related of encysted tumour of the thyroid gland, in which simple puncture produced no good effect, but the employment of a seton cured it. Dupuytren mentions two cases which were successfully treated by this remedy after some months; one, however, being diminished to two thirds of its size in seventeen days after the operation. On the whole he prefers it to the other surgical remedies, although the flow of venous blood is occasionally very great. Dr. Flajani, of Rome, likewise thinks it the safest manual remedy.

Accidental purulent discharges have been sometimes produced, as was the case of a blacksmith, which I have somewhere met with, who had a pendulous goitre reaching to his breast, and who, while working, severely wounded this tumour. A great quantity of viscid fluid, with clots and calcareous matter, escaped; the tumour subsided, and suppuration following, a speedy cure took place. Gautieri mentions a similar case of a Cretin who stabbed himself in this part.

The hemorrhage in cases where the seton is used is sometimes of alarming extent, however, and death has been known to follow from this cause. Tetanus has supervened occasionally on this operation, as instanced by Burns, Hedenus, and Beck.* It has been in many cases employed, too, without any benefit as to reduction of the tumour, though no symp-

* Über den Kropf.

toms of danger accompanied it. Dr. Monro used to mention in his lectures a case of bronchocele, combined with encysted tumour, in which a seton cured the latter, but not the former. Alibert, in a goitre of large dimensions, made use of it without any good effect. On the whole, however, it is an easier and safer operation than the two following, and may be resorted to in urgent cases, when a fair trial has been given to iodine without relief.

*Ligature of the Thyroid Arteries* was first performed in bronchocele by Mr. Blizard, in 1809; the tumor being much reduced in size after it, and promising a successful result, when gangrene carried off the patient. Walther, of Bonn, proposed it on the continent, in 1817; whilst Professor Waller, of Landshut, in 1818, and Mr. Coates, of Salisbury, in 1819, performed this operation with success. Mr. Earle, in 1822, published a very interesting case, in which he successively tied the right and left superior thyroid arteries, with great benefit to the patient. It will be found in the London Medical and Physical Journal for 1826. The great alterations of structure during the growth of the tumour, in some instances, render this operation extremely hazardous; causing great difficulty in reaching the vessels, and, as in the case of Mr. Key, at Guy's Hospital, where the operation occupied an hour, a fatal termination may arise from the great constitutional irritation which sets in.* Langenbeck, again, (though in his case the arteries, being superficial, were easily tied,) lost his patient thirty-four hours after the operation, owing to an anomalous distribution of the vessels, from repeated hemorrhages, though the carotid had been tied.†

This operation has been repeatedly since performed successfully, both in this country and abroad, and is a much safer proceeding than that of extirpating the gland. By the former we cut off a great supply of blood from the tumour, and thus gain time for using other remedies; and, should extirpation be at last deemed absolutely requisite to give the patient a chance for life, the difficulties of the latter operation may be lessened by this proceeding. The tumour in some cases diminishes much after the vessels are tied, but in the course of a few weeks regains its original size. This was the result of one related by Dr. Wells, of Colombia, in the American Journal of Medical Science.

*Extirpation of the Enlarged Gland* is one of the most difficult and arduous operations in surgery, as must be evident to everyone acquainted with the anatomical relation of

* Lancet, Vol. ii. † Neue Bibl. für Chirurg.

the parts. Above are the superior thyroid arteries, below, the inferior, all much enlarged in their diameter; in front a large plexus of veins; the carotids and jugulars at the sides, or even enclosed in the substance of the tumour; and behind, the recurrent nerves, par vagum, œsophageal plexus, trachea, and œsophagus: added to this, the multiplicity of open and bleeding vessels which require tying during the operation, will convince us that no surgeon, who is not an excellent anatomist, and endowed with great presence of mind, should undertake it. It has, however, according to Paradin and others, been performed solely to remedy the deformity occasioned by it; and the former author relates the fact of a barber who cut an enormous tumour off his wife's neck on this account; but this instance of temerity can never be quoted as an example. Numerous cases of fatal termination, some of which too were on the operating-table, are related by authors as following this dreadful operation. Petit gives us the case of a lady whose goitre interfered rather with her beauty than health, and who was persuaded to have the deformity removed, but died from hemorrhage. Palfin mentions another, of a lady of distinction, on whom the operation was performed by one of the most eminent surgeons of Paris, against the advice of the other practitioners consulted on the occasion: the lady resolved, however, to have it done, and begged them to be present; but, scarcely was the operation finished, when such an alarming hemorrhage came on, that, in spite of every attempt to restrain it, in a very short time it destroyed the patient; and, as Palfin observes, "au grande scandale de l'art, et du teméraire operateur qui contumier à de semblables enterprises n'eut d'autres excuses à alléguer, si non qu'il est toujours louable d'entreprendre les œuvres les plus difficiles, puisque le hasard seconde souvent ceux qui ont assez de courage pour tenter d'y réussir."*

Albucasis relates an instance where the patient died on the table from hemorrhage; and Pelletan, in his Clinique Chirurgicale, another, where a bronchocelous tumour of two pounds six ounces was extirpated; the operation occupying an hour and a half, and the patient dying in thirty-five hours after. Of Gooch's two cases, one died eight hours after the operation, and the other was with difficulty saved by constant pressure being kept up on the vessels for eight days by assistants. Klein had a patient die before him; whilst Major's and Dupuytren's cases terminated fatally from collapse.†

On the other hand, several eminent surgeons have pub-

* Anatomie Chirurgicale. † Journal of Foreign Med.

lished cases on which they had thus operated with very favourable results. Graefe having tied the thyroid artery in a girl of twenty-two, without any diminution of the tumour, she earnestly requested him to excise it altogether: he accordingly, in January, 1820, removed the right lobe, but was then obliged to desist, owing to the violence of the symptoms. In a few months after the girl returned to have the other lobe removed, which was done successfully. The tumour weighed two and a half pounds, and fifty-three ligatures were employed. Hedenus relates six cases in which he performed this operation successfully, but terms it "periculosa operatio."* Desault, Vogel, Theden, and others, likewise narrate cases ending favourably.

The consequent disturbance of the system alone, resulting from the long-continued manipulation among so many large vessels and nerves, must be attended with very great danger, which is heightened by the probability of air finding its way to the heart by some of the divided veins. Magendie relates a case of instant death from this cause; and I witnessed a similar one at the Hôtel Dieu, in which a tumour was removed by Dupuytren from the neck of a young woman, who fainted, as the spectators thought, in the middle of the operation, although she had scarcely lost three ounces of blood. On looking more closely, however, at her, life was found to be extinct; and, on opening the heart the morning after, Dupuytren and some of his assistants affirmed that they distinctly heard the escape of the air. Celsus says, speaking of this operation, "Sed scalpelli curatio brevior est," and details the steps of it; but so simple are they, that I am of opinion he never could have seen it performed. Boyer concludes the article upon this subject in his Traité des Maladies Chirurgicales thus: "L'extirpàtion de la thyroide est au nombre des opérations que la prudence, la raison, et l'experience désavouent."

Mr. Liston, of Edinburgh, in 1830, in one case of tumour connected with the thyroid gland, in which a seton, passed some time before through its centre, had rendered it more dense and immoveable, surrounded it by two semicircular incisions, and, dissecting cautiously beneath its base, detached it from its more loose connexions, without interfering with its central portion, and passed an armed needle through its centre, as near the trachea as possible, enclosing the remaining portions by the two ligatures, which removed them by sloughing: the hemorrhage was too profuse to allow his continuing longer with the knife. The patient was dismissed in six weeks, cured of all his previous distressing symptoms.

* De Gland. Thyr. Extirp., 1822.

Dr. Beck relates six cases of Struma Cystica, which he successfully treated by employing the following method. He laid bare the gland, and then removed several laminæ from it by the knife, so as to expose the cyst; an opening was made into this, and a portion of lint, covered with some stimulant application, inserted into it, so as to bring away the sac, &c. by suppuration. This is very similar, however, to a process recommended long since by Celsus, and more recently by Callisen. It would seem to be liable to the double dangers recounted as having followed both excision and the seton.

Simple incisions into a bronchocelous tumour have been sometimes followed by fatal results, as instanced by Flajani and other authors; and the application of caustic substances has, in similar manner, occasionally caused fearful sloughing and hemorrhage.

Too broad a line has, I think, been generally drawn between bronchocele and encysted tumours of the thyroid. Few of the former extend to any great dimensions without containing a cyst in some part of their substance, especially in accidental goitre: this arises from the rupture of several of its cells, gradually forming one large cyst, and is of course much more distinct when occurring near the surface. I have never seen one without an accompanying enlargement of the thyroid gland, and, in removing them by excision, we often find that the operators are somewhat embarrassed by their strong adhesions to the gland, and by the profuse hemorrhages which often follow this apparently simple operation. Incision is likewise frequently made into them to evacuate the contents, when a large venous discharge occasionally causes some alarm to the operator, lest he should have wounded one of the principal vessels; but this gradually stops, showing however the intimate connexion with the vascular and tumefied gland. On examining some of these tumours after excision, I have known them to consist not of one cyst, but of several cells, partly filled with clotted blood, and partly with a dark-coloured fluid.

The application of *Escharotics* to the external surface of the tumour is a proceeding fraught with much danger. The case related by Dr. A. T. Thomson of a woman who, on the recommendation of a friend, applied a plaster of quicklime to her bronchocele, is sufficient to point out the hazard attending this plan; though here fortunately the immense sloughing did remove the swelling, the patient narrowly escaping with her life.

Cautery was recommended by Paracelsus; but, if any surgical means are to be adopted when the disease absolutely requires them, either of the first two operations should certainly be tried; and, should they not succeed in putting a stop to the growth, the desperate, though sometimes successful operation of extirpation, is unavoidable, should the patient himself be anxious for it.

CASES.

I. February 21st. Miss A., æt. seventeen, of a leucophlegmatic temperament, but enjoying tolerably good health, perceived, six months since, a small swelling in the central and lower part of the throat, which gradually increased in size till within two months. At this period its progression was much more rapid. There is no pain on pressure in the tumour, which is externally about the size of a hen's egg, but more irregular in its form, extending laterally. Her breathing is much hurried on the least exertion; catamenia regular as to time, but not in quantity, inclining sometimes to menorrhagia.

She commenced taking eight drops of the iodine three times a day in a little water, and rubbing in a small portion of ointment, composed of ʒss. of Hydr. Potass. to ℥ss. of lard. In ten days the tumor was evidently becoming much smaller. The medicine was gradually increased in quantity, but discontinued again for some days, owing to its producing headach and other uneasy sensations. The ointment was afterwards used in the strength of ʒi. to the ℥i. In two months there was scarcely a vestige of the tumor to be seen.

II. August 15th. Mrs. C., æt. forty-five, a native of Shropshire, of healthy temperament, about twenty-four years since, when in an advanced state of pregnancy, received a severe fright from having seen the surgeon's hands covered with blood, after having performed some operation on her husband. She fell into an hysterical paroxysm, and a few days after first perceived a swelling in the throat: she almost daily had hysterical fits, sometimes to the number of eleven or twelve. At the end of three weeks she was confined, and the neck now presented a still greater fulness.

About twenty-one months after this she had a very protracted labour, and during it she perceived the neck to become unusually enlarged, and after this it continued slowly to increase. Two years since she had a miscarriage; the catamenia have not appeared since, but the growth of the tumour rapidly increased after their stoppage.

On examining the neck, a tumour, the size of a large orange, is seen exactly in its centre, of rather a soft nature, the left lobe being the part least affected. There is slight pain on pressure; respiration hurried after any exertion, and in damp weather. She is subject to giddiness and headachs, and hysterical feeling of suffocation about the throat supervenes on any agitation of mind.

September 1st. Six leeches were applied to the tumour, followed by a cataplasm and an aperient mixture. Immediately after the application of the leeches she felt faint, and the catamenia appeared after two years' absence. She commenced then with taking ten drops of the Solut. Hydriod. Potass. (℈ij. to the ounce,) three times a day, and using the ointment (ʒi. to the ounce.)

14th. Having increased the dose to twenty drops, without directions so to do, she in a few days complained of dragging pains in the stomach, great headach, giddiness, trembling, and depression of strength. Bowels costive, and great pain on passing fæces; blood sometimes accompanying them. Tumor less in size; pulse weak, but regular.—She was ordered to take an ounce of castor-oil; omit the drops; treatment continued.

18th. The foregoing symptoms nearly disappeared; tumour much reduced, having now the appearance of a central small lump, with lateral projections.

October 4th. Tumour still more diminished, but she finds a temporary enlargement occasionally from mental agitation. She was obliged to discontinue the ointment for some days, owing to a return of the symptoms, but has recommenced its use.

November 20th. Tumour now reduced to a very small lump, which does not incommode her.

The iodine seemed to affect this patient's constitution speedily, producing its bad effects occasionally; but as, from circumstances, she suffered much in mind at this period, that perhaps might have caused this predisposition.

III. February 19th. Thomas Cogswell, æt. thirty-one. Has a circular tumour at the front and lower part of the neck, of a firm nature, attended with much pulsation, and is about two inches in diameter, the right part being the larger. It made its appearance about two years since, but he can give no other history of it, as it did not cause pain or inconvenience. Pressure on it gives no pain, but much throbbing is felt after meals, or on lying down.—Hirud. tumori. Tr. Iodin. et Ung. Potassæ Hydriod.

March 8th. Has used the drops a fortnight, and the ointment for a week past, and the tumour is not so large as it was. He can now lie down without the disagreeable throbbing, nor does it come on after meals now.

18th. Tumour about the same size; complains somewhat of giddiness.

25th. Swelling much reduced in size; sleeps better than formerly.

This patient continued the use of the iodine, with occasional intermissions, till June, when the tumour had nearly disappeared.

IV. April 3d. Elizabeth Newberry, æt. twenty-one, native of Berkshire, of leucophlegmatic habit, says, that a month after her birth she was taken to a surgeon by her mother, on account of a swelling in her neck, and again when she was nine years old. It has gradually increased since that time. Does not enjoy good health. There is a general fulness of the throat between the sterno-mastoid muscles. The right side is the largest part of the tumour, extending nearly to the submaxillary bone upwards, and descending to the clavicle and sternum. On it, about half an inch from the sternum, is a small encysted lobule, the size of a chesnut. The swelling is tender on pressure at the left side, and the centre and right parts are so very painful that she cannot bear the least pressure with the finger. Respiration difficult, with choking sensation after meals; and she is unable to take any food which is hard, or requires much mastication. This same sensation, with violent throbbing, comes on when she lies down, and she is obliged to sleep with a very high pillow. Exertion distresses the throat much.

After applying leeches and a cataplasm, she commenced taking the Solut. Hydr. Potass., and using the ointment. The tumour became much reduced in size, and not so tender on pressure; she could eat meat, though not without difficulty; but she was not able to do so at all previously. She was attacked by an illness, which prevented her using the remedies for a time; but, on recommencing them, the tumour was still further reduced in size; but, as she was obliged to return to the country, I lost sight of her before it had disappeared.

v. May 12th. Matilda Green, ætat. forty-nine, a native of Berkshire, for the last six years has had a tumour, soft in its nature, forming on the right side of the neck; and, on examining it, the right lobe of the thyroid is found to have increased to the size of an apple. She mentions that her husband is similarly affected. Complains of difficulty in swallowing, but not in breathing: this has been the case for five months past, especially within the last week, and she is much troubled with giddiness. This patient used the solution only, without the ointment, and in the course of a month the tumour was very much diminished, as was the difficulty of swallowing.

vi. June 13th. Hannah Kennett, æt. twenty-two, native of Hanley, Worcestershire. Had always a full neck, but three years since a small tumour made its appearance in the situation of the thyroid gland, which has gradually increased since. It was at first painful; it is now the size of a small apple, occupying the whole of the gland, and of a soft texture, causing no uneasiness when touched, and not so lobulated as I have generally remarked this kind of swelling.—Ointment and drops.

24th. Much reduced in size.

July 4th. Still greater diminution. Has only used the ointment this last week. Whenever she catches cold she feels great uneasiness at this part, and can never wear anything tight round it.

14th. The tumour is nearly even with the surface of the neck. She remarks, that, when she lifts heavy weights, the throat feels very uneasy.

31st. Goes to Worcestershire tomorrow. Tumour scarcely to be felt.

VII. April 24th. Miss S., æt. sixteen, native of Colchester, caught cold two years since, and had ulcerated sore throat, with great difficulty of swallowing; she soon after observed a swelling on the central and right part of the throat, which gradually increased in size, and got round to the left side likewise. About six months since she came to London, and it has increased more rapidly during that time, the neck now measuring one and a half inches more than it did on her arrival. Has been occasionally much troubled with hysterics.

April 24. The tumour is at present the size of an orange, but larger at the right corner; it is not painful to the touch. It enlarges occasionally, when she catches cold, for a few hours, and generally is more full towards the evening; no difficulty in swallowing or breathing at any time, except when she catches cold.

May 11. Not much difference in the tumour, although she has been taking the drops, and using the ointment.

June 20. Tumour smaller and softer, but varies much more than I have ever observed in any other case: thus, a short time ago, in the morning, she found that it had decreased an inch in circumference since the preceding evening, and continued so for two days, when it as suddenly assumed its former size.

By persevering with the Hydr. of Potass. the circumference of the neck very materially diminished, as a letter from her in Colchester informed me, and she had completely lost that soreness which before used constantly to attend the slightest cold.

This patient's sister had likewise been affected with a bronchocele about the same size, which was removed by burnt sponge and local applications.

VIII. Mrs. E., æt. twenty-eight, native of Herefordshire. August 10th, 1829. About last Christmas she experienced a sensation in the throat as if she continually wanted to swallow. Taking a little brandy and water relieved this feeling much, and it disappeared at one time for a month; since which period, however, it has gradually got worse, as has the swelling about the part: in two months after this she began to experience shooting pains from the neck to the shoulders, up to the ears, and extending down the left breast under the clavicle, and they are now becoming worse. There is much throbbing on the right side of the neck after exercise. On lying down, she finds all the unpleasant symptoms increased, as they likewise are after meals, especially when she eats bread. Health pretty good, but the uterine function not regular, and she is much troubled by palpitation of the heart.

On examining the neck, there is a large flattened tumour occupying the situation of the thyroid gland, which is not very prominent, but follows the movement of the larynx in swallowing; it projects most on the right part, forcing out the sterno-mastoid muscle, under which it lies on either side. The right lobe varies in size, and she occasionally feels great numbness in the right arm, attended by a dark colour of the fingers. There is pain on pressure, and she sometimes experiences great sense of suffocation, principally when she goes long without food. No difficulty in swallowing. The size of the tumour, she says, is increased occasionally for an hour or two after dinner, and then subsides.

This case seems to present all the appearances of the *Goître en dedans*.—Hirudines vi. Solutionis Potassæ Hydriod. gtt. x. ter in die.

August 25. The swelling in the forepart of the neck is greatly reduced, and much softer. The sensation of swallowing, pain and uneasy feelings after meals, relieved in some degree. Throbbing and palpitations less. Catamenia very scanty this week.—M. xv. ter in die: to commence with ointment.

September 1st. Swelling still more diminished. The necessity of continually swallowing had disappeared for two days, but has since returned, though not to such extent as before: twenty drops.

8th. Within this last week great improvement; only the right lobe, in fact, is now to be felt, and that but slightly; the pain in left breast is much diminished; there is not so much throbbing; no numbness in the right arm; and lying down does not distress her. Passes occasionally two hours without any uneasy sensation in the throat. Pulse full; appetite very great. Complains of slight pain in stomach and tremulousness lately.—Omit the drops.

14th. Still uneasy feeling about the stomach, but, like other patients, cannot fully describe it: somewhat like hunger, but increased after meals.

October 8th. The uterine system is still disordered. At those times when the catamenia appear, though scantily, the throat is always more uneasy. There is now no appearance of tumour, but she still occasionally feels uneasy sensations about the part. The latter, however, disappeared likewise in the course of another month.

The four following cases benefited at the commencement under the employment of iodine; but, as I saw them at a public institution, and could not secure their regular attendance, I had not the opportunity of witnessing a removal of the tumour by its use.

IX. Elizabeth Trip, æt. eighteen. Has an enlargement of the thyroid gland, extending from the top of the sternum to the angles of the jaw. It has a soft doughy feel, and includes the whole of the gland. She has had a swelling at this part, she says, ever since she was a child, but it began to increase more rapidly when she was thirteen; there was no peculiar change at sixteen, when she menstruated. She is a native of Buckinghamshire (Bleadlow), and informs me that there are a great number of persons in those parts with similar tumours. This patient is of a healthy sanguine temperament, and the only inconvenience is from respiration being impeded occasionally, and from her *not being able to swallow any firm substance but with difficulty*.

x. Sarah Hutchinson, æt. thirty, has had a tumour as long as she can recollect in the front of the throat, but no inconvenience from it till after the birth of her first child, ten years since. She had a very severe labour, and tried to stifle her cries, as her husband was at the time lying very ill in the next room; œdema of the whole neck took place, which abated by next day, but the tumour began to increase more rapidly. Each succeeding labour augmented its size. After the fifth she found great inconvenience in breathing and swallowing. These symptoms have gradually increased, and at times respiration is nearly impeded altogether. The fifth child had great swelling of the neck at its birth, but it has since disappeared. This patient comes from Derbyshire, and mentions that the women there have to go a very long distance generally for water, the springs being on the hills, whilst the habitations are chiefly in the vallies; and she attributes this affection to having thus carried heavy weights daily on her head for miles. It is a curious fact, that she finds the tumour reduced somewhat in that county, and it increases again on coming to London. She has been subject to hysterics, and laughing, crying, &c. always produce difficulty of respiration. The tumour, which is of considerable size, appears distinctly lobulated, lying between the two sterno-mastoid muscles, with a clearly marked division of the central and two lateral lobes. Compression on the latter produces an immediate sense of suffocation.

xi. Hannah Esdaile, æt. twenty-nine. Has a small defined tumour of the right lobe of the thyroid, and occasionally experiences much shooting pains in it, especially on pressure; but respiration and deglutition are not affected. She first perceived it two years ago, after her first confinement, and it has gradually increased since, within this last fortnight especially. She is now three months advanced in pregnancy. Used the ointment only, and with good effect, as long as I had the opportunity of seeing her.

xii. Lydia Fawk, æt. twenty-five, native of Herefordshire, of a healthy constitution. About five years since perceived the front part of the throat to become more full than formerly: this increased gradually for a year, at the end of which time she removed to Olney, in Buckinghamshire, which she says lies damp and low, and where very many of the inhabitants are troubled with this disease. It now began to increase rapidly, and her breathing became much affected, attended with palpitations. The tumour occupies the central and left lobe of the thyroid, presenting a round elastic surface of a soft texture.

xiii. Mrs. J., æt. fifty-one, native of Sutton, in Hampshire. Has a large tumour completely in the centre of the neck, which is very soft to the touch. It made its appearance after the birth of her first child, and has gradually increased since. She has had two sisters likewise affected with it; one of whom, she says, had it whilst an infant, and it is now of very large size; the other, who is now seventy-four, has it still larger. Neither of them experiences any inconvenience from it. She does not know any other person in Sutton suffering under it. Another family who use the same well-water as themselves are not subject to the complaint. The solution of Hydriod. Potass. and the ointment had the effect of much diminishing the tumour in the course of a month.

For the following case I am indebted to the kindness of Mr. Maurice, of Marlborough. The preparation is in the Museum of Mr. Pilcher, and a cast of the tumour I forwarded to that of the College of Surgeons.

XIV. Lucy Osmond, at the age of eleven years, became the subject of bronchocele, and which increased for five years, though not to any material size; at the expiration of that time it grew very rapidly, and at eighteen exhibited the following appearances: Her head appeared buried in the tumour, which, from its size, and consequent pressure, threatened her with suffocation; her respiration was very laboured, and on the least exertion distressingly difficult; deglutition not impaired; headachs. The carotids are pressed on the outer part of the tumour, and pulsate strongly. The circumference of the whole neck is twenty inches, and the three lobes are distinctly marked. The tumour measures outwardly thirteen inches and three quarters, and projects beyond the sternum three inches, hanging an inch and a quarter below it. The tumour is still rapidly increasing, and respiration becoming more difficult; requiring some instant relief. The displacement of parts prevented the operation of tying the vessels, internally and externally. She commenced the use of iodine on November 3d, and in a fortnight great relief was obtained. At the expiration of three months the tumour had diminished an inch in every direction; and she continued the iodine for twelve months, with increased absorption of the tumour, and without detriment to her general health; at this period, however, it gave way; the long continued use of the iodine seeming to affect her powerfully. She became much emaciated, and the absorption of the tumour was now also wonderfully rapid. She died in April, eighteen months after commencing the before-mentioned treatment. An inspection of the tumour, or cast, will shew its great size, even at this time. Mr. Maurice has hardly ever found iodine to fail in common cases of bronchocele.

The following was obligingly forwarded to me by Mr. Miskin, of the Horseferry road. A cast of the tumour, which I sent likewise to the museum of the College of Surgeons, shows how small a size is sufficient to cause death, when extending behind the trachea.

XV. A poor woman, residing in the Kent-road, complained of the following symptoms: Great difficulty of breathing, with rising in the throat, attended with frequent desire of swallowing, as if she had some substance there which she could not get rid of. Frightful dreams disturbed her at night, and she had profuse perspirations at that time, attended with feelings of suffocation. Slight cough, white furred tongue, and costive bowels. Not confined to her bed more than a fortnight. Sixteen leeches were applied to the throat, and purgatives were exhibited three days afterwards; the leeches were repeated, and a blister was placed over the affected part. The tincture of iodine was then given, but on the fourteenth day she suddenly expired, with symptoms of suffocation.

The length to which this article has already extended prevents my inserting other cases, in some of which I did not find iodine produce any marked good effect, though continued for some time.

